# An Unsupervised kNN Method to Systematically Detect Changes in Protein Localization in High-Throughput Microscopy Images

**DOI:** 10.1371/journal.pone.0158712

**Published:** 2016-07-21

**Authors:** Alex Xijie Lu, Alan M. Moses

**Affiliations:** 1 Department of Computer Science, University of Toronto, Toronto, ON M5S3E1, Canada; 2 Department of Cell and Systems Biology, University of Toronto, Toronto, ON M5S3E1, Canada; Indiana University, UNITED STATES

## Abstract

Despite the importance of characterizing genes that exhibit subcellular localization changes between conditions in proteome-wide imaging experiments, many recent studies still rely upon manual evaluation to assess the results of high-throughput imaging experiments. We describe and demonstrate an unsupervised *k*-nearest neighbours method for the detection of localization changes. Compared to previous classification-based supervised change detection methods, our method is much simpler and faster, and operates directly on the feature space to overcome limitations in needing to manually curate training sets that may not generalize well between screens. In addition, the output of our method is flexible in its utility, generating both a quantitatively ranked list of localization changes that permit user-defined cut-offs, and a vector for each gene describing feature-wise direction and magnitude of localization changes. We demonstrate that our method is effective at the detection of localization changes using the *Δrpd3* perturbation in *Saccharomyces cerevisiae*, where we capture 71.4% of previously known changes within the top 10% of ranked genes, and find at least four new localization changes within the top 1% of ranked genes. The results of our analysis indicate that simple unsupervised methods may be able to identify localization changes in images without laborious manual image labelling steps.

## 1. Introduction

Advances in proteome-wide screening technologies [1] combined with high-throughput microscopy techniques have led to the development of image collections [2–4] where a large fraction of the proteome is tagged with GFP (green fluorescent protein) and systematically imaged, often across a number of chemical or genetic perturbations. As the localization of a protein provides important information on its regulation, identifying the proteins that change localization between a chemical or genetic perturbation and wild-type can characterize the systems-wide response of an organism to a perturbation. Thus, a major goal of these experiments is to systematically identify proteins that change in subcellular localization between screens and conditions, which we refer to as “change detection” for subcellular localization. Despite the importance of characterizing these localization changes, automated methods remain lacking: studies rely upon the manual assessment of localization over thousands of pairs of images between conditions [5,6]. Thus automated detection of localization changes is currently a key bottleneck in the discovery of key genes impacted by a perturbation.

Building upon successes of classification-based approaches in assigning localization to wild-type yeast proteins [7], previous work approached localization change detection in *Saccharomyces cerevisiae* through supervised learning, by defining sixteen morphological classes corresponding to subcellular localization classes and training classifiers from expert-curated examples from the wild-type [8]. Then, localization change detection is accomplished by comparing the classes assigned to wild-type genes versus the perturbation. By comparing a wild-type screen to a *Δrpd3* screen (a genetic perturbation), 31 protein localization changes induced by *Δrpd3* were found. However, we note several limitations in this method. First, the method is extremely time-consuming, requiring the manual curation of almost a hundred individual examples for each localization class (to be used as a training set for the classifier) representative of the range of variation in morphology, imaging, and intensity of markers. Second, it may not be easily generalizable, as all of the examples are curated from the wild-type; the model may not perform well on a perturbation that induces a change in cell morphology. Training a model for each experimental condition (in addition to the wild-type) is not desirable either.

The extensive training and problems with generalization for the supervised method motivated our development of an unsupervised method that acts directly on the image feature space. In this approach, we build a set of quantitative descriptors (features) to represent (profile) each gene, using measurements on the images. This is achieved by first segmenting images into single cells, and then extracting features (such as average intensity of GFP or statistics describing the spread of GFP) from each individual cell. The features corresponding to single cells can then be aggregated into features for each gene by averaging the features of all cells for a gene. Ljosa *et al*. [9] provide an evaluation of this method, in addition to several other methods of transforming single-cell features into gene features.

A naïve method for localization change detection is to simply subtract, for each gene, the features representing the gene in the perturbation from the features representing the gene in the wild-type (termed the “change vector”), which operates under the principle that if the genes are in similar localizations they should have similar feature values, resulting in values close to 0 where there is no change and highly positive/negative values where changes exist. However, this method is hampered by the presence of “global effects”, such as microscopy conditions or cell morphology changes, which lead to global changes in feature measurements, but do not necessarily affect all proteins and features in the same way.

To compensate for this issue, we employ a *k*-nearest neighbour (*k*NN) method. We retrieve the *k*-nearest neighbours of each gene in the wild-type feature space. Assuming the number of genes that change is likely to be small, the neighbours retrieved for each gene will be majority static (i.e. unchanged in localization in the perturbation relative to the wild-type); therefore, their changes will be representative of genes that are static and will capture the effects of global experimental differences in the local region of the feature space. We test if the change vector for a gene is an outlier compared to this set of change vectors to determine if a localization change has occurred. In principle, this method can be applied to any set of features. However, for the scope of this paper, we specifically demonstrate using a set of interpretable features that model the spread of protein within cells [10]. Overall, our unsupervised *k*NN method performs quite effectively relative to its simplicity and speed, capturing 71.4% of a list of previously-known localization changes ([8], for which we had enough data]) within the top 10% of ranked genes and facilitating the discovery of at least four new localization changes within the top 1% of ranked genes for the *Δrpd3* perturbation. Additionally, we demonstrate that the output of our method is useful for facilitating further manual assessment, generating a ranked list of genes with vectors that show the direction and magnitude of feature changes. The results of our analysis indicate that simple unsupervised methods may be able to identify localization changes in images without laborious manual image labelling steps.

## 2. Methods

### 2.1 Dataset

We use two screens from the publicly available CYCLoPs database for our experiments, rpd3del_1 (a RPD3 knockout screen) and its respective wild-type screen WT3 [4]. Four sets of micrographs per gene were retrieved from each screen, for 4143 genes.

### 2.2 Image Analysis and Feature Extraction

Image analysis and single-cell feature extraction was performed using the pipeline described by Handfield *et al*. [10], chosen due to its easily interpretable localization features. The pipeline first segments each image into single cells, and then extracts features from each cell based on the intensity and spread of GFP within the cell. For example, since the intensity of a GFP signal relates to the level of protein expression, the average distance of a protein to the centre of a cell can be estimated by weighing the distance of each GFP pixel to the centre of the cell by its intensity relative to the sum of intensities of all GFP pixels contained in the cell, and summing these values. Overall, the pipeline results in the assignment of a type (mother, bud, and lone) and six features describing localization (intensity of GFP signal, average distance between proteins, average distance to protein mass centre, average distance to cell periphery, average distance to cell centre, and average distance to bud neck) to each cell.

From these single-cell features, Handfield *et al*. generate features for each gene by binning the cells corresponding to that gene by size (10 bins) and by type (mother and bud, 2 bins) to represent cell cycle stage, before averaging single-cell features within each bin to generate a vector of 120 features. We follow this strategy with a small modification, where we reduce the number of bins for each type from 10 to 5 by merging adjacent bins. We do this to increase the number of cells within each bin, thus improving the reliability of the gene features. At this stage, we also filter the dataset under the criterion that a gene must have at least 5 cells in each bin for both the *Δrpd3* and wild-type screen in order to be retained. After filtering, 1985 genes remained.

As with Handfield *et al*., we compute the averages of each of the 6 features for all single cells in each of the 10 bins, resulting in a 60 feature vector for each gene in each the *Δrpd3* and wild-type screen. We note that using the mean of single-cell features sometimes results in gene features that are skewed by outlier cells in the bins (discussed more in the Results and Discussion section), so we also generate and benchmark two alternative profiles using the truncated mean (5% two-tailed cut-off) and the median, respectively, in place of the mean. Finally, gene features are rescaled to lie in similar ranges by dividing the intensity feature by 100 and multiplying localization features by 10 (as in Handfield et al.).

### 2.3 *k*NN Analysis and Ranking of Output

For each gene, we generate a change vector by subtracting the gene features of the *Δrpd3* screen from the wild-type. We chose a value of 50 for *k* from visually assessing the results of clustering of the wild-type gene features using hierarchical agglomerative clustering with average linkage and Euclidean distance, clustered using the Cluster 3.0 package [11]; from this assessment, we determined that the vast majority of genes appear to have at least 50 other genes with very similar localization feature patterns. We further validate this parameterization of *k* by evaluating values of 10, 25, 100, and 200 for *k*.

Thus, for each gene, we retrieve the *k* nearest neighbours in the wild-type gene features using Euclidean distance. To do this, we first calculate the pair-wise distance matrix, which stores the distance between each pair of genes based upon their profiles. For each pair of wild type gene feature vectors *a* and *b*, we calculate the Euclidean distance D(*a*, *b*), where *i* is the *i*th feature of the profile and *n* is the number of features in the profile:

$$D(a,b)=\sqrt{\sum_{i=1}^{n}{\left(a_{i}-b_{i}\right)}^{2}}$$

For each gene’s feature vector *a*, we look up the *k* smallest D(*a*, *b*) values and add each gene *b* to a set *B* representing the 50 nearest neighbours for *a*. Finally, we retrieve the change vector for each gene in *B* to create a matrix of *k* change vectors *X* of the *k* nearest neighbours, where x_a_ = a_wt_−a_RPD_

We then want to compare the change vector for the gene of interest to this matrix of change vectors to determine if the gene is an outlier in its change or not. We do this in a feature-wise manner: for each feature in the gene’s change vector, we calculate the modified z-score M based upon the mean and the median absolute deviation of the *k* values for that feature in the set of change vectors:

$$M_{i}=\frac{0.6745(x_{i}-{\tilde{x}}_{i})}{{MAD}_{i}}$$

Where *i* is the *i*th feature, such that *x*_*i*_ is the *i*th feature of the gene’s change vector and ${\tilde{x}}_{i}$ is the median of the set of change vectors for the gene’s neighbours for the *i*th feature, and *MAD*_*i*_ is the median absolute deviation of the set of change vectors for the gene’s neighbours for the *i*th feature:

$${MAD}_{i}={median}_{im}(|x_{im}-{median}_{in}(x_{in})|)$$

Where *x*_*i1*,_ *x*_*i2*_ *… x*_*ik*_ are the features of the set of change vectors for the gene’s neighbour for the *i*th feature. By repeating this for each feature in each gene, we calculate a vector of modified z-score corresponding to the features for each gene.

We consider more strongly positive/negative modified z-score as stronger outliers and therefore, as an indicator of a more likely or dramatic localization change. We use the modified z-score as it is robust against outliers [12]–while we can assume the set of change vectors largely reflects static genes due to the relative rarity of localization changes, localization changes may still occasionally be represented. Overall, our *k*NN method is related to *k*NN regression, with two key differences. First, rather than simply using neighbours to predict the conditional expectation for a point, we detect deviation from the conditional expectation using the z-scores; second, the neighbours are chosen based on the similarity of the gene profiles associated with each point, and the z-score (which includes the *k*NN conditional expectation) are computed based on the change vector.

From running this procedure, the output of the *k*NN analysis is a vector of 60 modified z-scores for each gene, each corresponding to a gene feature. To isolate localization changes, we take the root mean square (RMS) of the modified z-scores for only the 50 localization features for each gene; we use RMS to smooth outliers (as a strong modified z-score in just one gene feature may be an error caused by outlier single cells or other factors) while still up-weighing strongly positive/negative z-scores. For each gene, the RMS is calculated as:

$$RMS=\sqrt{\frac{1}{n}\sum_{i=1}^{n}{M_{i}}^{2}}$$

Where *i* is the *i*th feature, *n* is the total number of features, and *M*_*i*_ is the modified z-score for the *i*th feature.

Genes are ranked by order of descending root mean square modified z-score value.

### 2.4 Evaluation of Rankings

To obtain a measure of precision, we compare the output of our *k*NN method to the 31 known genes with localization changes originally discovered by Chong *et al*. [8]. Within our filtered 1985 genes, 14 of these genes are retained. We note that Chong *et al*. appear to be able to apply their classifiers to much more of the data; this can be explained by the features used and the filtering strategies employed in our respective experiments. Whereas we choose features that bin cells by size to capture cell cycle time-points, Chong *et al*. initially pool all cells together and use a classifier to identify 3 cell cycle points. Thus, while we explicitly filter by sample size at this stage, this task is handled implicitly within tests that Chong *et al*. employ to determine if a localization change is significant or not. To visualize our results, we plot the fraction of known localization changes found against the fraction of all genes in the dataset retrieved (by rank cut-offs). Additionally, we retrieve the micrographs corresponding to the top ranked genes by our *k*NN method for the mean profile and qualitatively assess these micrographs with the assistance of the modified z-score vectors of the corresponding genes to determine localization changes.

Finally, we compare our results against a naïve method using just the change vectors. We rescale features of the change vectors by subtracting the mean and dividing by the sample standard deviation of the feature for all change vectors. Then, we calculate the RMS of the change vector for each gene, and rank the results using RMS (as described in the previous subsection).

### 2.5 Visualization of Results

We visualize our matrixes of gene profiles, modified z-scores, and change vectors using heat maps generated by Java Treeview [13]. In these visualizations, rows represent genes and columns represent features. Features are ordered first by type (bud, then mother), then by bin (1 to 5), then by feature in order of intensity, average distance between proteins, average distance to protein mass centre, average distance to cell periphery, average distance to cell centre, and average distance to bud neck (abbreviated in figures as INT, SEF, MCT, EDG, CEN and NEC, respectively). Colour represents sign of value (green being negative, red being positive), while intensity of colour represents magnitude (with a brighter colour being stronger).

## 3. Results and Discussion

### 3.1 *k*NN Compensates for Cluster-Specific Global Changes

**Fig 1:**
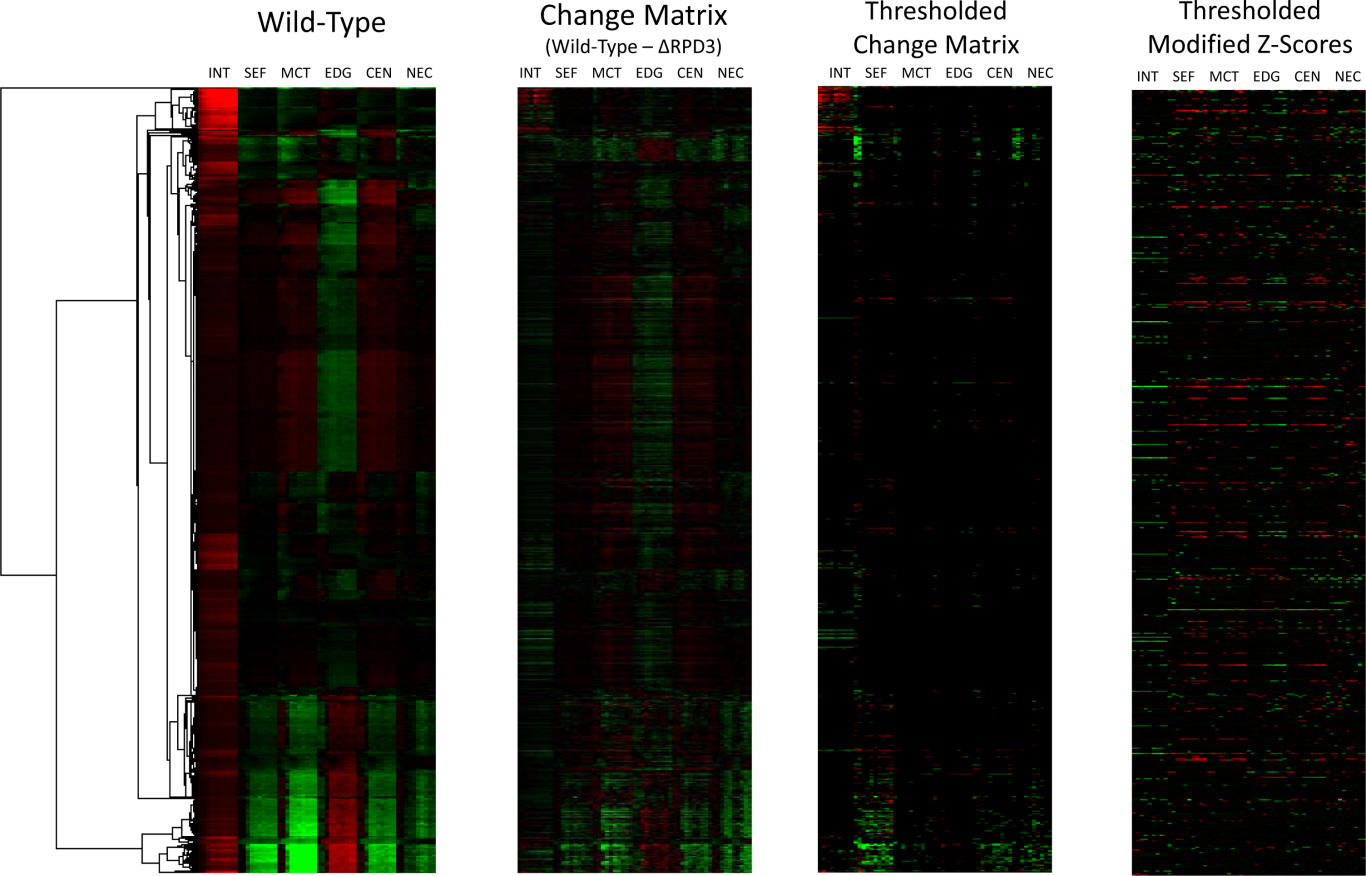
Heat map visualizations of the naïve method versus our *k*NN method. Clustered wild-type gene features, the change matrix, the thresholded change matrix, the naïve method (features thresholded using a cut-off of absolute feature > 12.0), and the thresholded modified z-scores from the *k*NN method (features thresholded using a cut-off of absolute feature > 2.0) are shown from left to right. Information on the heat map visualization can be found in the “Visualization of Results” section in the Methods.

Retrieving and visualizing the change vectors for our clustered wild-type gene features shows that the change matrix exhibits systematic effects that are consistent within clusters, but differ between clusters. Additionally, some clusters exhibit more dramatic effects in terms of magnitude. The uneven impact of global changes upon clusters means simply looking for large magnitudes in the change matrix is unreliable. As we show in Fig 1, thresholding the change matrix simply results in the highlighting of genes in subcellular localizations disproportionately impacted by global effects. In contrast, the thresholded modified z-scores show a spread of values across subcellular localizations, suggesting the *k*NN method successfully compensates for the uneven global effects across subcellular localization clusters.

### 3.2 Benchmarking of Ranked *k*NN Genes with Known Changes

**Fig 2:**
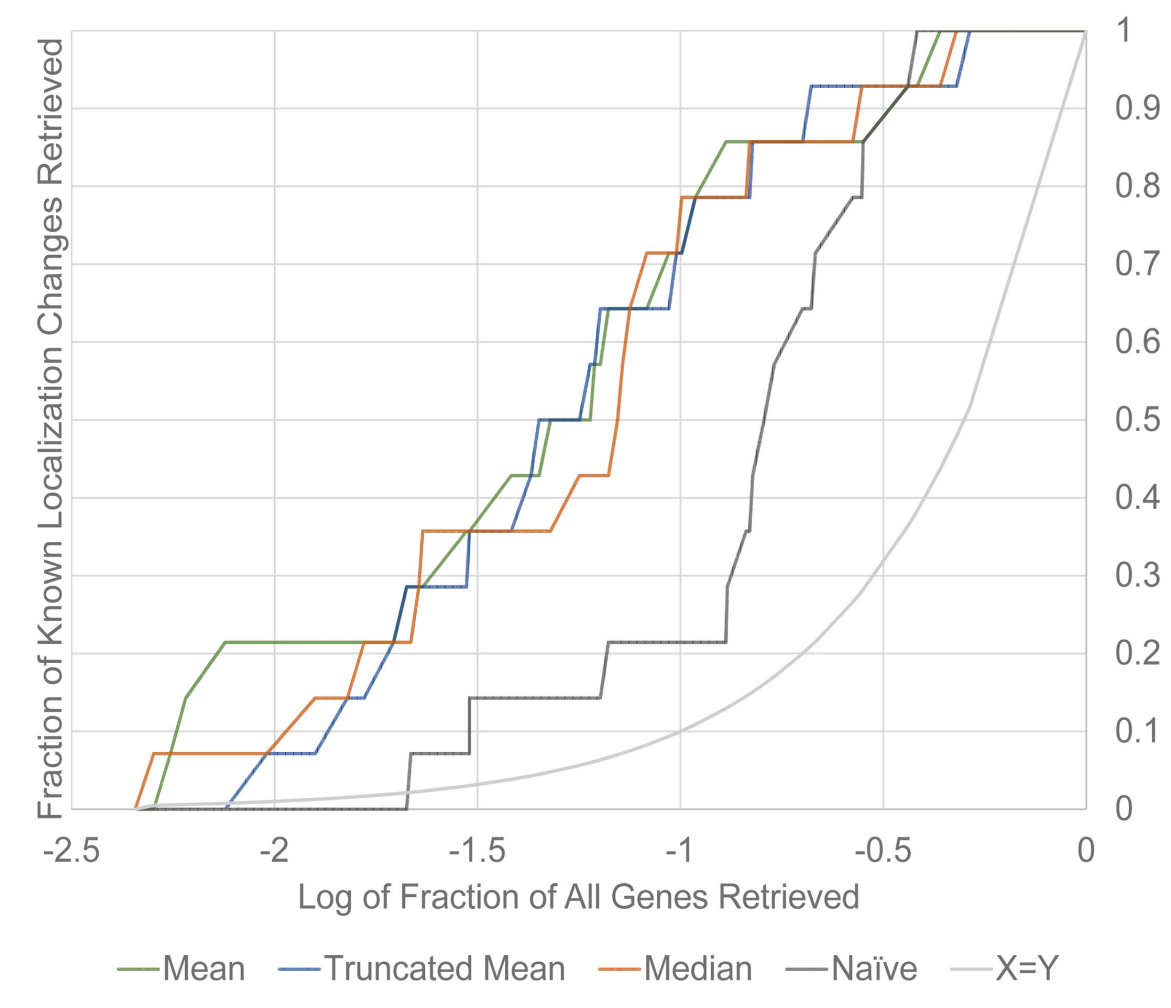
Fraction of known localization changes retrieved against the log_10_ of fraction of all genes retrieved for the *k*NN method with mean, truncated mean and median profiling. We also show the naïve method and the X = Y curve for comparison.

We plot the fraction of known localization changes retrieved against the logarithmic-transformed fraction of all genes in the dataset retrieved for the naïve method compared to the *k*NN method using various methods of profiling genes (Fig 2), using the genes found with the supervised method by Chong *et al*. [8]. We caution against taking these plots as an exact indicator of precision as the unsupervised algorithm may be sensitive to different localization changes compared to Chong *et al*.’s supervised method. For instance, since our *k*NN algorithm implements thresholds and robust measures to reduce noise, it may not be sensitive to smaller changes in localization. Conversely, as we demonstrate in our qualitative assessment of our top 20 ranked results, we find some localization changes through our *k*NN method not found previously. However, the comparison still provides a rough indication of precision–for instance, we can see that the *k*NN method for all profiles clearly outperforms the naïve method up until at least 85% of known localization changes retrieved.

**Fig 3:**
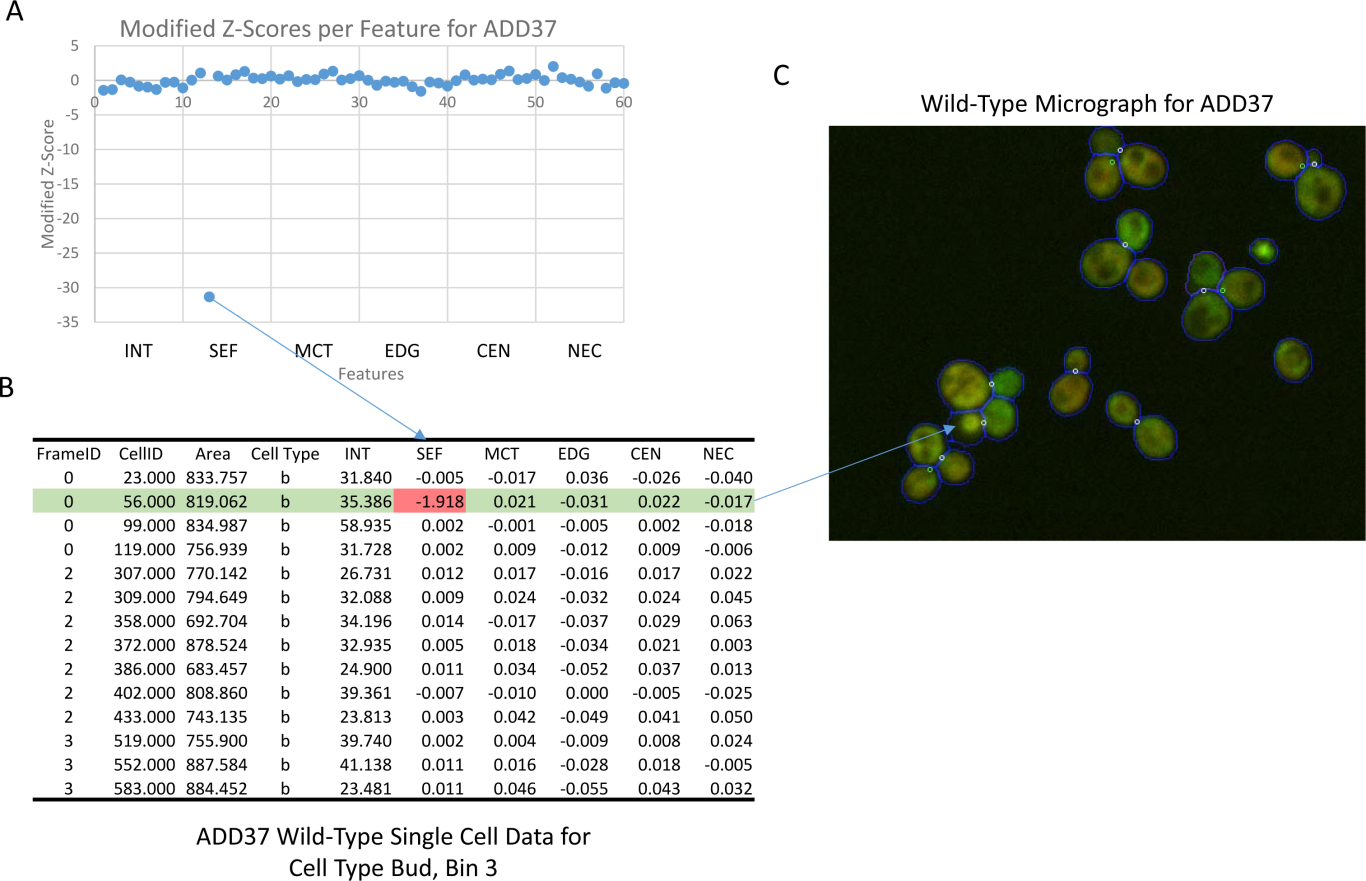
Example of a false positive generated by outliers in single-cell data. ADD37 is reported as a top-ranked gene for localization change using mean profiles despite no obvious phenotype. A shows the feature for distance between proteins is very strongly negative in bud cells in bin 3. The single cell-data for this bin is shown in B, which shows a single cell has a disproportionately high value for this feature, skewing the mean. Looking up this cell in the micrograph in C shows that the cell is either mis-segmented or expressing the protein differently from other cells. Feature abbreviations can be found in the “Visualization of Results” section in the methods.

Originally, we used the mean profile for genes. However, we noted that this measure was sensitive to outliers in the single-cell data, occasionally generating highly ranked genes that showed no discernible localization change. For instance, as shown in Fig 3, for the gene ADD37, the modified z-scores show a highly negative value in the distance between proteins measure of bud cells in the third bin. Evaluating the wild-type single cell data identified a single bud cell for that bin with either a highly abnormal morphology or an error in segmentation. However, because this cell had a much lower distance between proteins feature relative to the other cells in the bin, it ended up heavily affecting the average for this feature relative to the *Δrpd3* feature.

To make our method more robust to single-cell outliers, we implemented a truncated mean and a median profile for genes. However, this represents a trade-off. From qualitative assessment of the top 20 hits, we observe that many of the genes identified to have a localization change still have a large portion of cells in the *Δrpd3* micrographs with the same subcellular localization as the wild-type micrographs, with the localization change obviously affecting the proportion of cells localizing to a compartment or producing new subcellular localization classes not witnessed in the wild-type (or vice-versa). The truncated mean and median profiles are less sensitive to these changes. While the performance of the truncated mean and median later becomes comparable to the mean, we consider that in the most straightforward practical use of retrieving images for qualitative assessment, we are primarily concerned with the top few hits to narrow down genes for further assessment. Thus, we assess the top genes ranked by the mean for qualitative evaluation, in spite of its greater sensitivity to single cell outliers, in part 3.4 of this paper.

We also note that the naïve method outperforms the *k*NN method in retrieving the final ~7% of known localization changes, suggesting there are certain types of localization changes that are difficult for the *k*NN method to discover. To an extent, this behaviour is expected. First, we note that the naïve method still does capture localization changes to an extent, with the key issue being the extremely large number of false positives due to noise and global effects; in other words, the naïve method still performs better than random chance. Second, one limitation of our method is that it may generate false negatives where multiple proteins exhibit identical localization changes. For these genes, neighboring genes in the wild-type gene features may have similar change vectors, reducing the magnitude of the modified z-scores. While we do not observe this specific phenomenon for this screen, we observe a similar one: the lowest ranked of the known positives was CGR1, which was previously found as a nucleus to nucleolus localization change by Chong *et al*. [8] As genes localizing to these compartments are not very well separated by our features, the variance in the distribution of change vector for the static nucleus-localized neighbors may have overlapped with the change vector of CGR1, de-emphasizing the subtle changes for this gene.

### 3.3 Parameterization of k is a Balance

**Fig 4:**
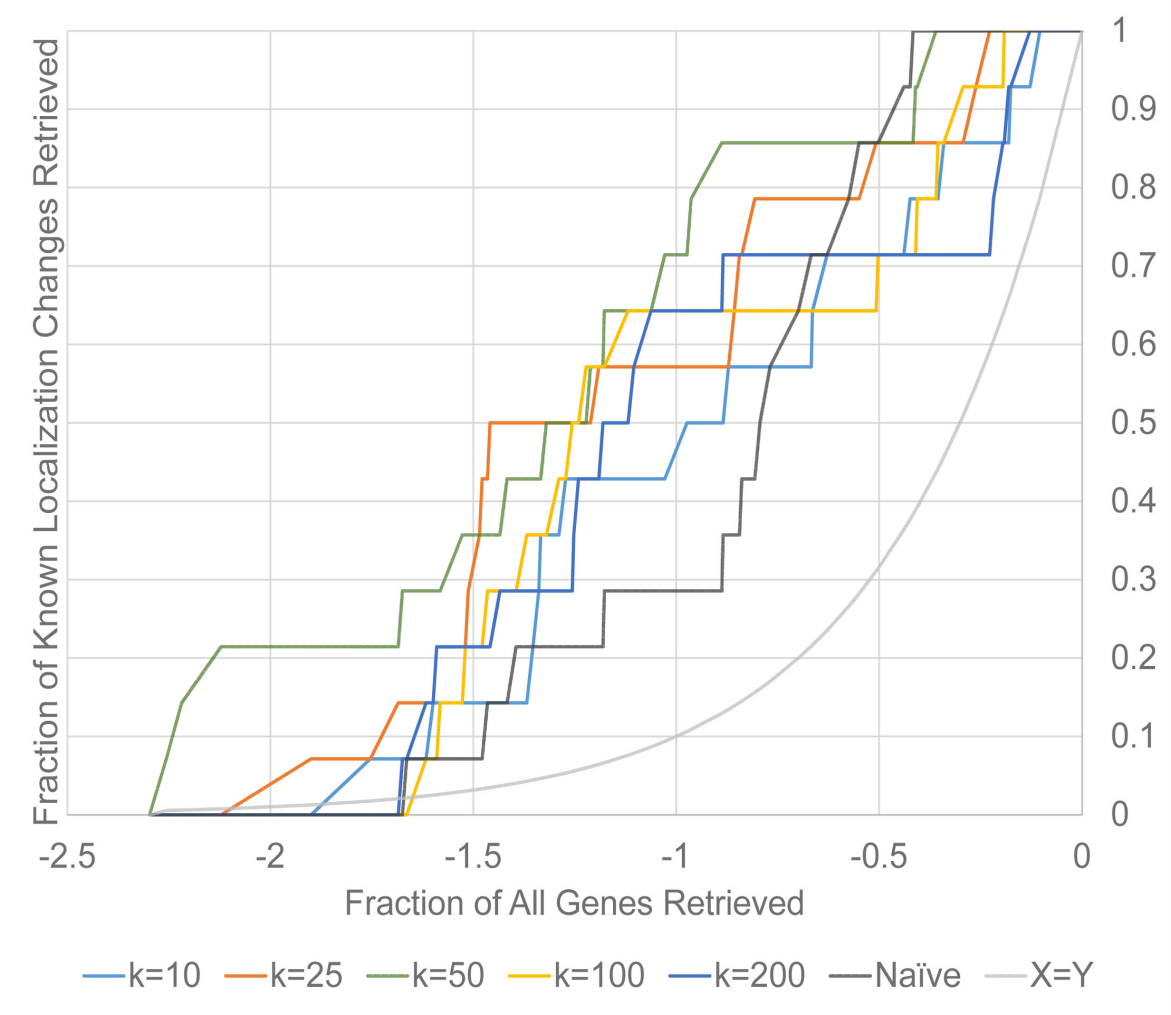
Fraction of known localization changes retrieved against the log_10_ of fraction of all genes retrieved for the *k*NN method for *k* = 10, 25, 50, 100 and 200, respectively. We also show the naïve method and the x = y curve for comparison.

To validate our selection of *k* = 50, we tested the performance of the algorithm with several other parameterizations of *k* (10, 25, 100, and 200). We visualize these results in Fig 4 as plots of fraction of known localization changes retrieved against the logarithmic-transformed fraction of all genes in the dataset retrieved for the naïve method compared to the *k*NN method, using the mean profiling method for all plots.

In general, we observe the best performance from *k* = 50. We also observe that *k* = 10 performs worse than *k* = 25, and *k* = 200 mostly performs worse or comparably to *k* = 100. Together, these results suggest that there is an optimal point for *k*. Too low of a *k* parameter may result in inadequately-powered change vector comparison sets, and may result in false negatives for genes that change in localization together. But too high of a *k* parameter may cause false positives from the value of *k* exceeding the number of genes that localize to some compartments in the wild-type, resulting in the retrieval of a change vector comparison set that includes genes from other localization classes. However, the differences between the various selections of *k* used in this experiment are fairly subtle, suggesting the method is robust.

### 3.4 Qualitative Assessment of Top Ranked Genes

**Fig 5:**
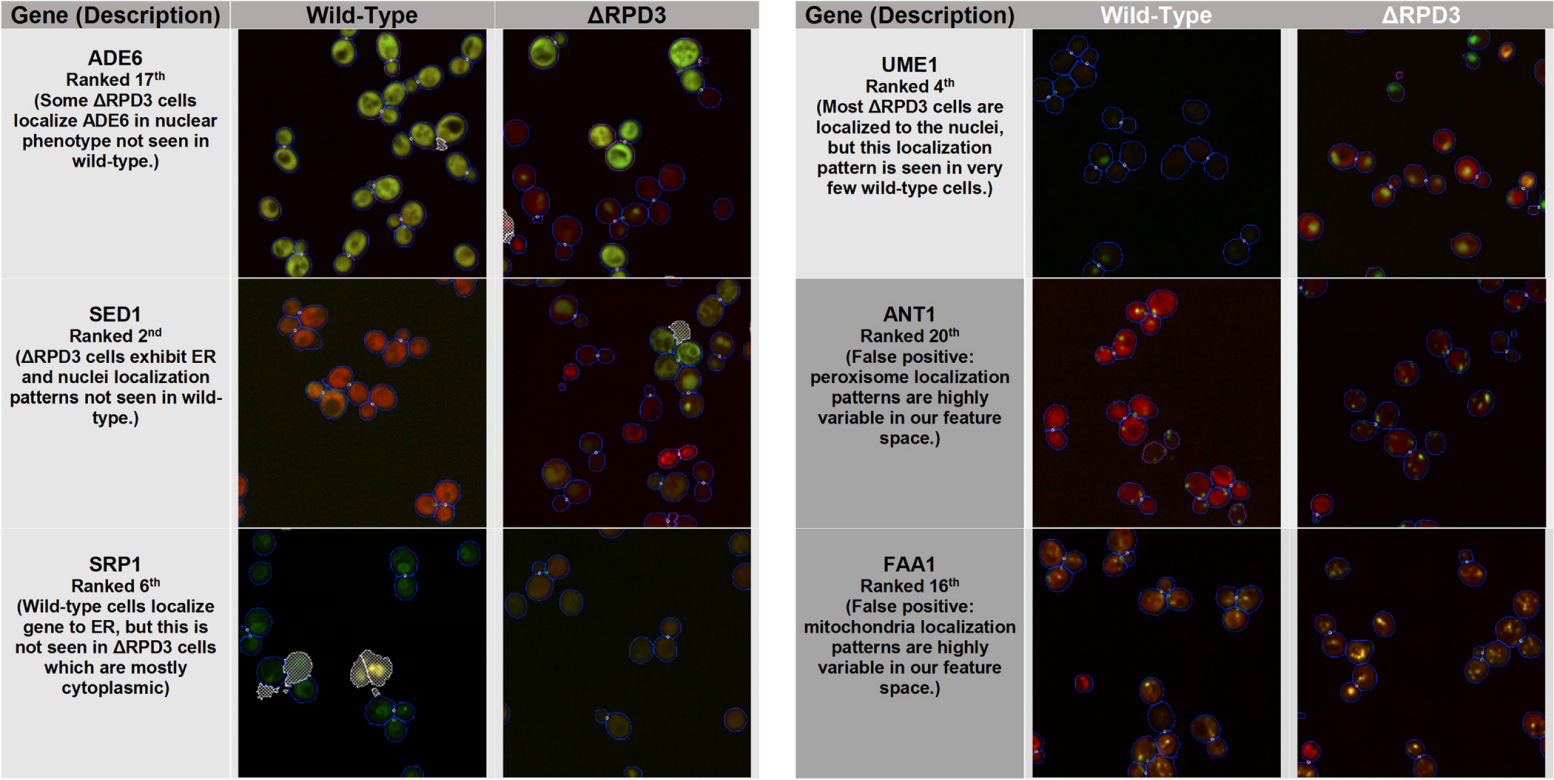
Curated examples from the top 20 ranked genes from the mean profile. Representative images from the wild-type and *Δrpd3* screens are shown for 4 newly-found genes exhibiting localization changes, along with a description the change and the rank of the gene using the mean profile. In addition, we show two examples of false positives in the top 20 ranked genes. Cell segmentations are outlined in blue, with mother-bud associations shown as white circles. The white cross-outed regions are artifacts discarded by the image analysis software.

From the top 20 genes ranked by our *k*NN method using the mean profile for k = 50, we identify several new localization changes in addition to 3 previously found by Chong *et al*. (PAB1, RAD7, and YCR061W) [8]. We show 4 examples of new changes found by our method in Fig 5 (ADE6, SED1, SRP1, and UME1), along with a description of the localization change. These examples represent only the most visually obvious localization changes within the top 20 genes; not all examples are easily validated by human eye, as they may contain more subtle changes such as shifts in proportion of cells in each localization class. Thus, while the lower bound on the number of new changes found in the top 20 genes ranked by our method is 4, there may be further localization changes contained.

In addition to the false positives caused by outlier cells described in the previous subsection, we note that the *k*NN method is sensitive to localization classes that are highly variable in morphology (such as the mitochondria, peroxisome, or the endoplasmic reticulum), and to genes that are variably expressed (e.g. temporally expressed genes). Some examples are documented in Fig 5. We suggest that part of this sensitivity is due to the feature selection. As Handfield *et al*. note, while their features have the advantage of being easily interpretable, they do not necessarily outperform more complex features [10]. To filter subcellular compartments with highly variable morphology, our *k*NN method may benefit from the inclusion of features or use of feature sets that account for this variability (e.g. texture measurements) in order to reduce the distance of the change vector between the *Δrpd3* perturbation and the wild-type in the feature space relative to “real” localization changes. We note that previous unsupervised approaches for tracking protein localization across the cell cycle in time-lapse microscopy have used texture features in distinguishing some localization changes [14].

### 3.5 Modified Z-Score Vectors are Interpretable

**Fig 6:**
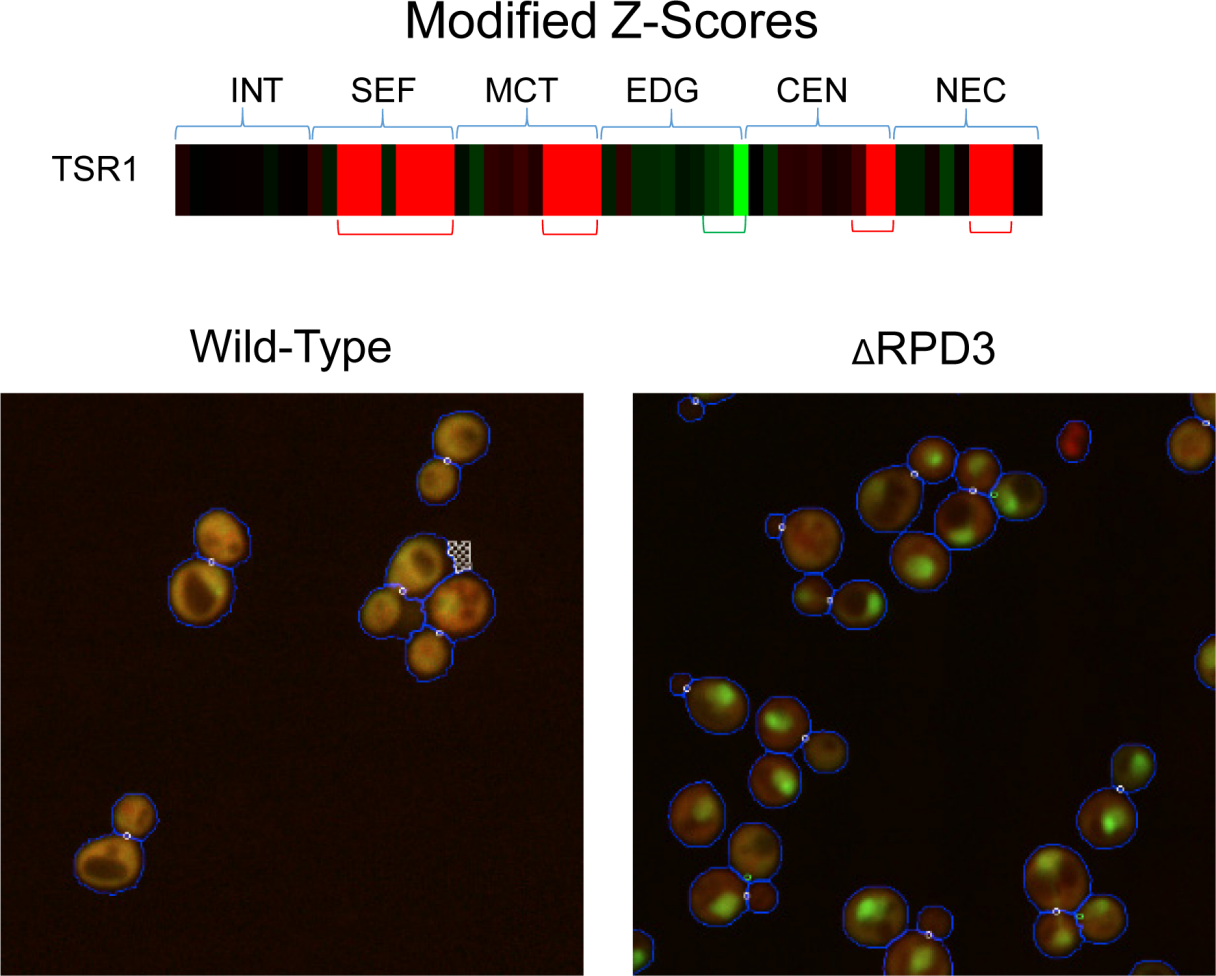
Modified z-score vectors can be interpreted to determine direction of feature-wise change. We show the modified z-score vector for TSR1 as a heat map (more information in “Visualization of Results” section in the Methods) and representative images of TSR1 for the wild-type and the *Δrpd3* screen; TSR1 has strongly positive modified z-score in mother features for distance between proteins, distance to protein mass centre, distance to mass centre, and distance to bud neck, and negative ones in features for distance to cell periphery. Thus, we expect the former features to be larger in the wild-type than the *Δrpd3* and the latter to be larger in the *Δrpd3* than in the wild-type. In other words, we expect the GFP in *Δrpd3* mother cells to be denser, closer to the nuclei, closer to the bud neck, and further from the cell periphery relative to the wild-type. This interpretation is consistent with the localization patterns seen in the images.

Owing to the interpretable properties of the features selected for these experiments, the modified z-score vectors can be used to interpret the nature and direction of the average localization changes of the sample. The sign of the modified z-score of a gene feature indicates the direction in which we expect the feature to deviate from a gene that remains static; a negative modified z-score indicates that we expect the change vector feature to be smaller, whereas a positive modified z-score indicates that we expect the change vector feature to be larger. The change vector is the wild-type gene features subtracted from the *Δrpd3*; thus, a positive value in a feature indicates that the gene feature is larger in the wild-type than the *Δrpd3* profile, and a negative value indicates the opposite. We demonstrate in Fig 6 for the localization change for the gene TSR1 (ranked 22^nd^ by mean profile), which is also newly discovered by our method owing to its high placement in our rankings.

## Conclusion

In this paper, we demonstrate a simple but surprisingly effective unsupervised method for the proteome-wide detection of localization changes. The key advantage of our method is that it is extremely fast to operate compared to previous supervised attempts at this problem, requiring only the feature space and no expert labelling of images. In addition, the method generates a quantitatively ranked list of changes along with a modified z-score vector that represents the feature-specific directions of change, facilitating manual qualitative assessment and providing the potential for further refining and processing of output.
